# Use of the Monocyte-to-Lymphocyte Ratio to Predict Diabetic Retinopathy

**DOI:** 10.3390/ijerph120810009

**Published:** 2015-08-21

**Authors:** Song Yue, Jiahua Zhang, Jingyang Wu, Weiping Teng, Lei Liu, Lei Chen

**Affiliations:** 1 Department of Ophthalmology, The First Affiliated Hospital of China Medical University, No.155 Nanjing North Street, Heping District, Shenyang 110001, China; E-Mails: yuesong567@163.com (S.Y.); jiahuaz1986@163.com (J.Z.); happygirl860824@163.com (J.W); 2 Department of Endocrinology and Metabolism, Institute of Endocrinology, Liaoning Provincial Key Laboratory of Endocrine Diseases, The First Affiliated Hospital of China Medical University, Shenyang 110001, China; E-Mail: twp@vip.163.com

**Keywords:** platelet-to-lymphocyte ratio, monocyte-to-lymphocyte ratio, neutrophil-to-lymphocyte ratio, diabetic retinopathy

## Abstract

*Background*: Diabetic retinopathy (DR) is a common complication of type 2 diabetes mellitus (T2DM) and the leading cause of blindness in adults. DR pathogenesis has not been fully elucidated, but inflammation is widely accepted to play an important role. Emerging evidence suggests that the platelet-to-lymphocyte ratio (PLR), monocyte-to-lymphocyte ratio (MLR), and neutrophil-to-lymphocyte ratio (NLR) are novel potential markers of inflammatory responses. The present study aimed to evaluate the associations between DR and the PLR, MLR, and NLR. Patients and *Methods*: We performed a case-control study involving 247 patients with T2DM. The patients were divided into three groups: 125 control subjects with T2DM, 63 diabetic subjects with non-proliferative diabetic retinopathy (NPDR), and 59 patients with proliferative diabetic retinopathy (PDR). *Results*: The mean PLR and NLR were significantly higher in patients with DR compared with patients without DR (*p* < 0.01, *p* = 0.02, respectively). The mean MLR in the NPDR group was higher than that of patients without DR, but there were no significant differences among the three groups (*p* = 0.07). Logistic regression showed that the MLR was an independent risk factor for DR (odds ratio [OR]: 54.574, 95% confidence interval [CI]: 2.708–1099.907). Based on the receiver operating characteristic (ROC) curve, use of the MLR as an indicator for DR diagnosis was projected to be 2.25, and yielded a sensitivity and specificity of 47.1% and 69.6%, respectively, with an area under the curve of 0.581 (95% CI: 0.510–0.653). *Conclusions*: The PLR and NLR are significantly increased in the setting of DR. After correcting for possible confounding factors, the MLR was found to be a risk factor for DR. Although the MLR may be pathophysiologically and clinically relevant in DR, its predictive ability was limited.

## 1. Introduction

Many reports indicate that Asia will become the epicenter for diabetes mellitus (DM), particularly in developing countries [1,2]. As DM prevalence increases, so does the development of diabetic retinopathy (DR). DR is a serious complication of DM, and is considered a major cause of blindness in the working age population [3]. DR pathogenesis is complicated and related to many factors, but many groups have described the role of inflammatory factors in DR development [4,5,6,7].

The white blood cell (WBC) count and its subtypes are classic indicators of inflammation [8]. The platelet-to-lymphocyte ratio (PLR), monocyte-to-lymphocyte ratio (MLR), and neutrophil-to-lymphocyte ratio (NLR) are potential markers of inflammation in various conditions, including tumors [9,10,11], cardiovascular conditions [12,13] and other diseases [14].

To date, only a few articles have studied the relationship between diabetic microvascular complications and the PLR, NLR, and MLR [15,16,17,18]. Some studies have reported that the PLR and NLR are associated with diabetes and its complications [15,19]. Akbas *et al.*, reported that the PLR and NLR were significantly elevated in diabetic nephropathy patients with albuminuria [15]. The associations of the PLR and MLR with DR have not been investigated to date. Although the relationship between DR and the NLR has been studied, the results were contradictory. Ulu *et al.* [17] reported a positive correlation between the NLR and different DR grades but Ciray *et al.* [16] failed to find an independent association between the NLR and DR. The present study aimed to evaluate the relationships between DR and the PLR, NLR, and MLR.

## 2. Patients and Methods

### 2.1. Study Population

The case-control study was conducted from August 2014 to April 2015 in the Fengyutan health care center, Shenhe District, Shenyang City, China. Additional details were described previously [20]. We assessed a total of 246 patients diagnosed with T2DM, 121 of whom had DR. Patients were excluded if they were younger than 18 years old or had type 1 diabetes mellitus, any acute inflammation, active infection, cancer, chronic liver diseases, or any diabetic microvascular complications except DR.

### 2.2. Clinical Examination and Biochemical Analysis

All the subjects underwent stereo fundus photography to detect DR using a 45° Non-Mydriatic Fundus Camera (CR6-45NM, Canon, Tokyo, Japan) [21,22,23] through undilated pupils. For every subject, two fundus images centered on the fovea and optic disc for each eye were taken in a darkened room. Each image was independently graded in a masked manner by two well-trained ophthalmologists from Liaoning Diabetic Eye Center for DR diagnosis. If the grades were different, a third ophthalmologist would reach the final decision. The DR grade for each eye was determined, and an individual’s classification was based on the worse eye.

Participants’ systolic and diastolic blood pressures (SBP and DBP) were measured after a 5-min rest using a sphygmomanometer. The mean values of two measurements were recorded. Venous blood samples were drawn after an overnight fast. All biochemical analyses were performed in our hospital, including fasting plasma glucose (FPG), total cholesterol (TC), triglyceride (TG), low-density lipoprotein cholesterol (LDL), serum creatinine (Scr), blood urea nitrogen (BUN), high-density lipoprotein cholesterol (HDL), and glycosylated hemoglobin A1c (HbA1c).

### 2.3. PLR, NLR, MLR, DM, and DR Definitions

The NLR, PLR, and MLR were calculated as the ratios of the neutrophils, platelets, and monocytes to lymphocytes, respectively. All counts were determined from the same automated blood sample measurement. DR was evaluated according to the International Clinical Diabetic Retinopathy Disease Severity Scale [24]. Diabetes was diagnosed according to the 1999 World Health Organization criteria [25].

### 2.4. Statistical Analysis

All data were expressed as a median and interquartile range (IQR) because of non-normally distributed numerical variables. Non-parametric analyses were used to compare the data. We performed chi-square, Kruskal-Wallis H and Mann Whitney U tests for dichotomous and continuous variables, respectively. Logistic regression was used to analyze DR risk factors. Receiver operating characteristic (ROC) curve analysis was used to compare the prognostic powers of the PLR and MLR for DR. The predictive validities were quantified as areas under the ROC curves. The positive predictive value (95%CI) and negative predictive value (95%CI) were calculated by MedCalc ver 15.2.1 (MedCalc Software, Mariakerke, Belgium). Others analyses were performed using SPSS 17.0 (SPSS for Windows, version 17.0; SPSS, Inc., Chicago, IL, USA), and *p* < 0.05 was considered statistically significant.

## 3. Results

**Figure 1:**
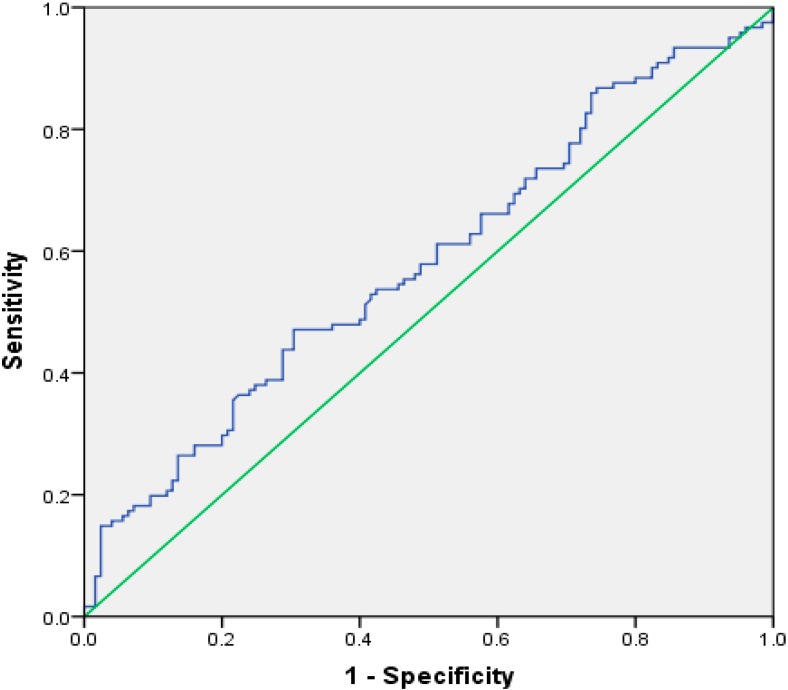
Receiver–operating characteristics (ROC) curve analysis for monocyte to lymphocyte ratio as a predictor of the severity of diabetic retinopathy.

The patients were divided into three groups: 125 control subjects with T2DM, 62 diabetic subjects with NPDR, and 59 patients with PDR. The baseline characteristics of all 246 subjects are shown in Table 1. We observed significant differences for DM duration, SBP, DBP, HbA_1_c, TC, HDL-C, LDL-C, PLT, NLR, PLR, and MLR (*p* < 0.05). DR patients were divided into two groups based on severity (NPDR and PDR). The results are shown in Table 2. The PLR and NLR were significantly higher in patients with DR compared with patients without DR (*p* < 0.01 and *p* = 0.02, respectively). The mean MLR of patients with NPDR were higher than those of patients without DR (median [IQR] 0.22[0.30–0.14] *vs.* 0.18[0.24–0.13]), but there were no statistical differences among the three groups (*p* = 0.07). In addition, retinopathy severity was not associated with increased NLR, PLR, or MLR. Logistic regression analysis revealed that independent risk factors for DR were DM duration, SBP, MLR, TG, HDL-C, and PLT (Table 3). Figure 1 shows that as an independent risk factor for DR, the cut-off value of MLR was 2.25, and the sensitivity and specificity of the MLR for DR diagnosis were 47.1% and 69.6%, respectively, with an area under the curve at 0.581 (95% confidence interval (CI): 0.510–0.653). The positive predictive value (95%CI), negative predictive value (95%CI) for MLR for DR diagnosis were 59.82% (49.25% to 69.75%) and 57.80% (49.5% to 65.78%), respectively.

**Table 1:** Patients’ baseline and clinical characteristics.

Variables	DM (*n* = 125)	DR (*n* = 121)	*p*
Age (years)	56.00[63.00–48.00]	55.00[61.00–47.00]	0.23
Sex			
Male (%)	73 (58.4)	62 (51.2)	0.31
Duration of DM (years)	5.00[10.00–1.50]	10.00[16.00–7.00]	<0.01
Family history of DM			
Yes (%)	47 (37.6)	49 (40.5)	0.69
SBP (mmHg)	125.00[140.00–110.00]	140.00[150.00–130.00]	<0.01
DBP (mmHg)	80.00[80.00–70.00]	85[90–80]	<0.01
FBG (mmol/L)	8.00[11.00–6.00]	9.20[12.00–6.50]	0.09
HbA1c (%)	7.00[9.00–6.00]	8.00[10.00–7.20]	<0.01
TG (mmol/L)	2.00[2.50–1.00]	1.43[2.17–1.00]	0.93
TC (mmol/L)	5.00[5.50–4.00]	5.00[5.86–4.00]	0.03
HDL-C (mmol/L)	1.15[1.79–0.80]	2.20[3.21–1.00]	<0.01
LDL-C (mmol/L)	3.00[4.00–2.00]	1.53[3.00–1.00]	<0.01
Scr (umol/L)	59.00[70.00–51.00]	58.00[74.00–46.00]	0.69
BUN (mmol/L)	6.00[7.00–5.00]	6.09[7.90–5.00]	0.18
WBC (×10^9^/L)	6.25[7.55–5.37]	6.51[7.87–5.39]	0.37
Lymphocytes (×10^9^/L)	2.10[2.65–1.61]	2.00[1.53–2.41]	0.13
Neutrophils (×10^9^/L)	3.52[4.57–2.90]	3.88[4.85–3.0.2]	0.13
Monocytes (×10^9^/L)	0.37[0.46–0.29]	0.38[0.30–0.55]	0.19
Platelets (×10^9^/L)	197.00[239.50–161.50]	210.00[255.00–182.00]	0.02
NLR	1.74[2.29–1.31]	1.99[2.62–1.47]	0.02
PLR	94.04[120.19–70.73]	107.75[149.82–84.44]	<0.01
MLR	0.18[0.24–0.13]	0.20[0.29–0.14]	0.04

Data are expressed as median (inter-quartile range) or percentage; DBP: diastolic blood pressure; SBP: systolic blood pressure; FBG: Fasting blood glucose; HbA1c: Hemoglobin A1c; TG: triglycerides; TC: total cholesterol; HDL-C: high-density lipoprotein cholesterol; LDL-C: low-density lipoprotein cholesterol; Scr: serum creatinine; BUN: blood urea nitrogen; WBC: white blood cell; NLR: neutrophil-to-lymphocyte ratio; PLR: platelet-to-lymphocyte ratio; MLR: monocyte-to-lymphocyte ratio.

**Table 2:** Comparison of inflammatory and hematologic parameters in patients with and without retinopathy and its severity.

Variables	DM (*n* = 125)	NPDR (*n* = 62)	PDR (*n* = 59)	*p*-Value
Age (years)	56.00[63.00–48.00]	53.50[60.25–46.00]	56.00[62.00–50.00]	0.17
Sex				
Male (%)	73 (58.4)	34 (54.8)	28 (47.5)	0.38
Duration of DM (years)	5.00[10.00–1.50] ^#,†^	10.00[15.00–6.75] *,†	15.00[19.00–8.00] *,#	<0.01
Family history of DM				
Yes (%)	47 (37.6)	28 (45.2)	21 (35.6)	0.50
SBP (mmHg)	125.00[140.00–110.00] ^#,†^	135.00[145.00–122.00] *,†	145.00[150.00–130.00] *,#	<0.01
DBP (mmHg)	80.00[80.00–70.00] ^#,†^	84.00[90.00–80.00] †	85.00[90.00–80.00] #	<0.01
FBG (mmol/L)	8.00[11.00–6.00]	9.10[12.34–6.91]	9.35[14.00–6.46]	0.25
HbA1c (%)	7.00[9.00–6.00] #	8.00[10.00–7.00]	8.20[10.00–7.40] #	0.02
TG (mmol/L)	2.00[2.50–1.00]	1.70[2.16–1.00]	1.41[2.21–1.00]	0.53
TC (mmol/L)	5.00[5.50–4.00]	5.00[6.00–4.00]	4.99[5.46–4.00]	0.12
HDL-C (mmol/L)	1.00[1.00–1.00] #,†	1.00[2.49–1.00] *,†	2.86[3.46–2.00] *,#	<0.01
LDL-C (mmol/L)	3.00[4.00–2.00] #,†	2.50[4.00–1.09] *,†	1.24[2.00–0.99] *,#	<0.01
Scr (umol/L)	59.00[70.00–51.00]	59.00[71.25–46.00]	57.00[78.75–45.75]	0.99
BUN (mmol/L)	6.00[7.00–5.00] #	6.00[7.00–4.85] *	6.75[9.99–5.06] *,#	0.01
WBC (×10^9^/L)	6.25[7.55–5.37]	6.67[8.11–5.41]	6.43[7.50–5.34]	0.42
Lymphocytes (×10^9^/L)	2.10[2.65–1.61]	2.03[2.36–1.49]	1.97[2.43–1.52]	0.29
Neutrophils (×10^9^/L)	3.52[4.57–2.90]	3.88[4.93–3.07]	3.75[4.60–2.85]	0.16
Monocytes (×10^9^/L)	0.37[0.46–0.29]	0.39[0.56–0.31]	0.38[0.53–0.28]	0.27
Platelet (×10^9^/L)	197.00[239.50–161.50] #	202.00[236.25–177.00]	225.00[264.00–182.00] #	0.03
NLR	1.74[2.29–1.31] #,†	2.05[2.75–1.55] †	1.91[2.51–1.39] #	0.03
PLR	94.04[120.19–70.73] #,†	105.07[151.42–81.53] †	115.73[145.97–87.98] #	<0.01
MLR	0.18[0.24–0.13] †	0.22[0.30–0.14] †	0.20[0.28–0.15]	0.07

Data are expressed as median (inter-quartile range) or percentage; DBP: diastolic blood pressure; SBP: systolic blood pressure; FBG: Fasting blood glucose; HbA1c: Hemoglobin A1c; TG: triglycerides; TC: total cholesterol; HDL-C: high-density lipoprotein cholesterol; LDL-C: low-density lipoprotein cholesterol; Scr: serum creatinine; BUN: blood urea nitrogen; WBC: white blood cell; NLR: neutrophil-to-lymphocyte ratio; PLR: platelet-to-lymphocyte ratio; MLR: monocyte-to-lymphocyte ratio; ^*^ Significant difference between PDR and NPDR; ^#^ Significant difference between DM and PDR; ^†^ Significant difference between DM and NPDR.

**Table 3:** Logistic regression analysis showing independent predictors of retinopathy.

Variables	OR	95%CI	*p*-Value
Duration of DM	1.162	1.085–1.246	<0.001
SBP	1.033	1.013–1.054	0.001
MLR	54.574	2.708–1099.907	0.009
TG	1.671	1.026–2.722	0.039
HDL-C	7.357	3.004–18.017	<0.001
LDL-C	0.625	0.359–1.091	0.098
Platelet	1.007	1.000–1.013	0.043

OR: Odds Ratio; 95% CI: 95% confidence interal; SBP: systolic blood pressure; MLR: monocyte-to-lymphocyte ratio; TG: triglycerides; HDL-C: high-density lipoprotein cholesterol; LDL-C: low-density lipoprotein cholesterol.

## 4. Discussion

As a serious microvascular complication of DM [26], DR is a complex disease involving multiple events. Powell *et al.*, previously reported that some anti-inflammatory drugs such as salicylates could prevent the occurrence of DR, suggesting that inflammation may play a role in DR pathogenesis [27]. Lutty *et al.*, verified the association between white blood cells and DR occurrence [28]. Moreover, some epidemiological studies have indicated that DM and its microvascular complications are associated with chronic inflammation [29,30] and immune responses [31,32,33]. There are common sets of inflammatory cytokines and immune biomarkers including tumor necrosis factor (TNF)-a, interleukin (IL)-6, IL-1β, and intracellular adhesion molecule (ICAM)-1 that are up-regulated in both serum and vitreous fluid in subjects with DR. These findings suggest an interaction between inflammation and DR pathogenesis [34,35].

As important inflammation response markers, counts of WBCs and its subtypes are associated with cardiovascular disease [8]. Besides WBC counts, the PLR, MLR, and NLR are potential biomarkers reflecting inflammation and immune responses. Many studies have reported positive correlations of conventional inflammatory markers with the PLR and NLR. More importantly, a large number of studies found predictive effects of the PLR and NLR, particularly in DM, acute coronary syndromes, and various cancers [9,10,12,13,36]. However, as an immune indicator, the only study on MLR focused on malaria [14].

Our results suggest that DM patients with DR had higher NLRs compared with diabetic patients without DR. This is in accordance with the findings of Ulu *et al.* [17] and Wang *et al.* [18]; however, it was contrary to the results reported by Ciray *et al.* [16]. They found that the NLR was not significantly different in patients with or without DR. In addition, our results are different from those of Ulu *et al.*, who found that DR severity was not associated with increasing NLR. The discrepancy between these two studies might be due to different sample sizes, as our cohort was slightly larger. Subject heterogeneity and lifestyle difference may be other reasons for the discrepancies. Notably, the NLR has been shown to reflect an immune microenvironment that favors vascular invasion by tumors [37]. Therefore, we have reason to believe that there is a correlation between the NLR and DR.

As stated above, PLR and MLR have been studied in many fields. This is the first investigation of the relationship between DR severity and the PLR and MLR, which are viewed as new inflammatory markers. Our results indicate that the PLR and MLR were significantly higher in patients with DR compared to those without. Some angiogenesis factors such as vascular endothelial growth factor (VEGF) are key protein modulators expressed by platelets [38]. Notably, high VEGF levels can stimulate the development of proliferative diabetic retinopathy (PDR). This would suggest that there is association between the PLR and DR progression.

Furthermore, there was no correlation of either the PLR or MLR with DR severity. Akbas *et al.* [15] showed that the PLR could predict inflammation and albuminuria in patients with diabetes. Hudzik *et al.*, reported that the PLR was an independent risk factor for early and late mortality in patients with DM [36].

We only found one study investigating the relationship between the NLR and DR. The results suggested that higher NLR values may be a useful marker for DR [17]; however, they did not use ROC analysis to assess the NLR predictive performance. In our study, the NLR was not an independent risk factor for DR, so we did not perform ROC analysis to determine its predictive value for DR. The most important implication is that although the MLR correlated with the presence of DR and was an independent risk factor, it performed poorly as a screening tool for DR diagnosis. Monocytes are considered a biomarker for inflammation because their activation leads to the synthesis of inflammatory cytokines. A previous report suggested that monocytes may be relevant to angiogenic processes in atherosclerosis [39]. Nevertheless, the mechanisms underlying the association between the MLR and DR should be investigated in future studies.

As novel markers to predict DR, the PLR, NLR and MLR are superior to other cell parameters, e.g., neutrophil, lymphocyte, and total leukocyte counts. The advantage is that the PLR, NLR, and MLR are the absolute value counts and show good stability even when physiological, pathological, and physical factors of the WBC count vary. In addition, they may represent inflammatory and immune signaling in DR.

Our study has some potential limitations. First, the case-control study design did not allow us to investigate whether controlling the MLR may affect DR outcome. Second, we assessed a relatively small number of subjects. Third, we did not perform fundus fluorescein angiography (FFA) to detect DR.

## 5. Conclusions

In conclusion, the PLR and NLR are significantly increased in the setting of DR. Although the MLR is an independent risk factor for DR in Chinese patients with T2DM, it has a low performance for DR diagnosis. Additional studies are needed to identify mechanisms explaining the association between the MLR and DR.
